# Study on mechanical properties and energy change of rock materials in whole splitting process based on peridynamics

**DOI:** 10.1038/s41598-023-30394-5

**Published:** 2023-02-24

**Authors:** Hewan Li, Jian Liu, Laigui Wang, Tianjiao Ren

**Affiliations:** College of Mechanics Engineering, Liaoning Technical University, No. 47, Zhonghua Road, Xihe District, Fuxin City, 123000 Liaoning Province China

**Keywords:** Civil engineering, Energy infrastructure

## Abstract

Aiming to bypass the inability to directly observe the evolution process of rock internal deformation and fracture, this paper proposes that rock samples with different inclination angles can be analyzed from the standpoint of energy, using the bond-base peridynamic theory and the PMB model of brittle materials, combined with laboratory experiments. The whole process of shearing is analyzed, and the LAMMPS software is used to simulate the internal energy change of rock-like materials under shear conditions, while the damage evolution law of samples with different dip angles is studied from macro and micro perspectives. The result shows that prefabricated cracks and the inclination of cracks are important factors for specimen damage, a finding that has important theoretical value for rock mechanics research. The research results can reduce the occurrence of rock burst accidents, the difficulty of mine support, and the cost of mining engineering, as well as improve mine safety levels.

## Introduction

Due to the increase in the depth and breadth of mining, the working face of the roadway gradually moves, resulting in various types of defects such as pores, cracks, and joint structures in the rock mass^1^. Sandstone is a typical rock type around underground energy. Under the influence of the above defects, its mechanical properties change, which directly affects the difficulty of mine support and other projects and can even lead to disasters such as rock bursts^2,3^. Therefore, it is urgent to study the mechanical properties of sandstone and the laws of crack propagation during loading.

Tensile failure is one of the most basic failure modes of rock materials. It can be used not only to calculate a series of important physical parameters, such as rock tensile strength, but also to study the crack propagation and macroscopic fracture properties throughout the whole process of rock shear^4^. Various crack propagation laws can be realized by prefabricating cracks on rocks and changing crack angles^5,6^. Based on this, many scholars across the globe have carried out analyses on the process of shearing various rock-like materials using the Brazilian splitting experiment. Xiong et al.^7^ used shale as the experimental material, combined with a self-made "herringbone"-shaped mold to measure the fracture toughness at two crack angles, and obtained the law of shale fracture toughness. Shang et al.^8^ studied the effect of pressure on the fracture toughness of disc specimens with prefabricated cracks, and the results showed that when the specimen contains large-angle cracks, the load pressure needs to be analyzed. When Zhu et al.^9^ used RFPA software to numerically simulate the whole process of rock material shear with prefabricated cracks, the analysis showed that the prefabricated crack will guide the damage in the rock material when the specimen is in contact with the loading device and near the crack tip. Likewise, H. Haeri et al.^10^ performed shear experiments on disc specimens with prefabricated single and double cracks by combining experiments and numerical simulations. In this way, the number of cracks and the damage law of angular rock materials was found. These existing shear experiments mainly focus on the single analysis of crack propagation and material macroscopic damage, and most of them are based on fracture toughness and crack propagation law. However, few studies examine the crack propagation along with the change of internal energy during the whole shearing process of the material; in the above numerical simulations, the finite element analysis method is mostly used, but the mesh divided by the finite element in the simulation process will have a certain influence on the solution accuracy. In addition, in the finite element solution process, the differential equations used are often discontinuous and the position partial derivatives do not exist, which leads to difficulties in analysis and errors in the analysis results^11,12^.

Peridynamics (PD theory), as a theory of nonlocal modeling, describes the mechanical properties of brittle materials by integrating equations such as those for motion. It does not need divided grids, and cracks can expand freely^13,14^. At the same time, PD theory also includes a variety of analysis methods such as bond-based PD theory, which effectively avoids the above-mentioned discontinuous solution problem and shows high solution accuracy in both macro and micro situations^15,16^. Therefore, in this paper, complete and prefabricated sandstone materials with different dip angles are used for shear experiments to analyze the crack orientation and damage of the samples. The PD damage constitutive force function is deduced using the unique advantages of PD theory in calculating energy. According to the constructed constitutive force function model, a numerical simulation is carried out in the LAMMPS software to explore the change of the model’s internal energy during shearing and compare and analyze it with the experimental results to study the whole process of rock shearing damage. The research results aim to provide a reference for reducing the occurrence of rock burst accidents and difficulty of mine support, thereby reducing the cost of mining engineering and improving the mine safety level.

## Indoor shear experiment of rock specimen

**Figure 1:**
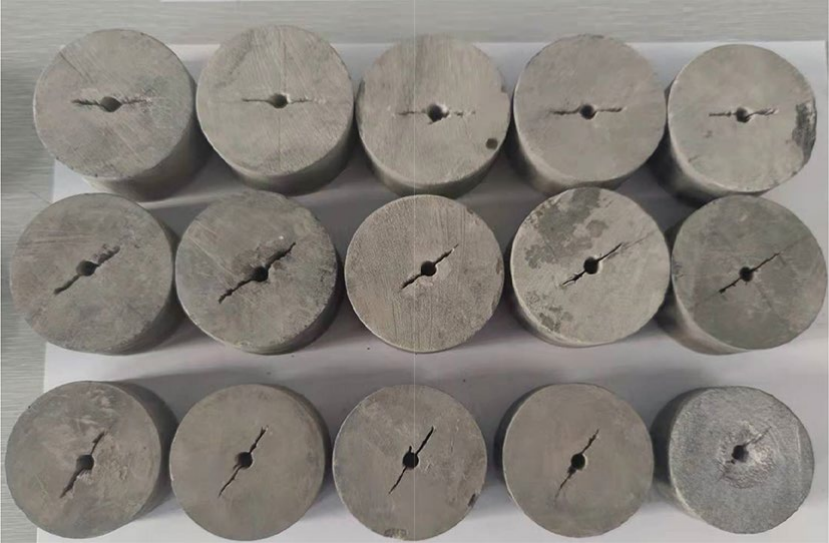
Partial model diagram and loading diagram.

Fine sandstone was selected as the experimental material, and the rock was prepared into a cylindrical sample of *Φ*50mm × 25 mm according to the standard recommended by ISRM. The actual measurement would prevail, and the error was less than 0.2 mm. In addition, referring to the method of prefabricating cracks by Chen^17^, the sample was drilled in the center first, and then saw wire was used to process cracks with different inclination angles, using a crack width of about 0.35 mm. Some of the prepared samples are shown in Fig. 1, and different sample numbers are shown in Table 1.

**Table 1:** Sample numbers of different rock types.

Class	Sound specimen	0°	30°	60°
Fine sandstone	XS	XS-0	XS-30	XS-60

**Figure 2:**
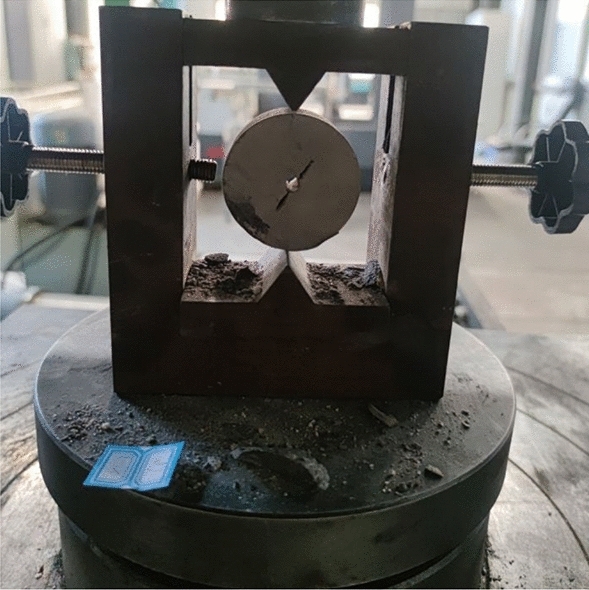
Disc shear loading method.

The fine sandstone was subjected to shear tests using a universal tensile test machine and a Brazilian splitting mold. The loading method is shown in Fig. 2. The tensile strength of the samples was then measured and the propagation law of cracks in the samples was analyzed. The loading displacement was 0.1 mm/min. Each lithology sample was tested five times, and the optimal result was selected as the experimental result.

### Institutional review board statement

“Not applicable” for studies not involving humans or animals.

## Experiment results and analyses

**Figure 3:**
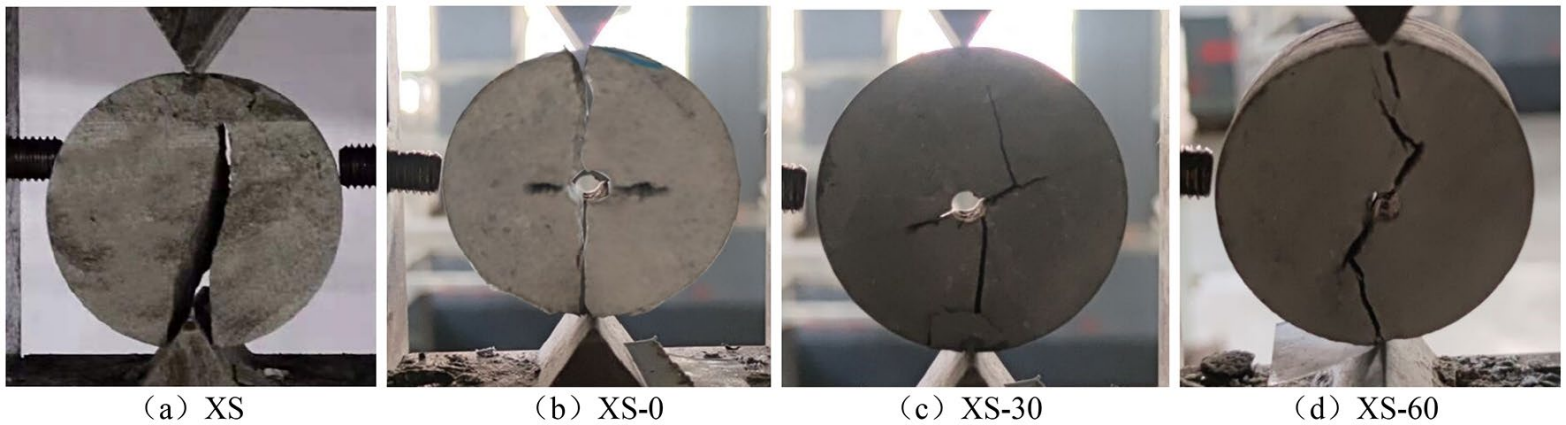
Shear results of disc specimens.

According to the experimental plan, the shearing experiment was carried out on four groups of disk samples, and the failure results of different samples are shown in Fig. 3.

In Fig. 3, it can be seen that the crack propagation morphology of different samples is different during the shearing process. For the XS specimen, although only the upper and lower tip parts were stressed, the crack propagation still deviates from the loaded position. Compare this with the XS-0 sample: when this specimen was sheared, the crack propagation was approximately a straight line, and the propagation direction was close to the upper and lower loaded tips. With the increase of the prefabricated crack angle, a crack was gradually generated at the position of the crack tip, which was approximately perpendicular to the prefabricated crack, and then gradually connected to the upper and lower loaded tips. This shows that the prefabricated crack has a great influence on crack propagation during the whole shearing process of the specimen. The larger the inclination angle of the prefabricated crack, the more obvious the effect.

**Figure 4:**
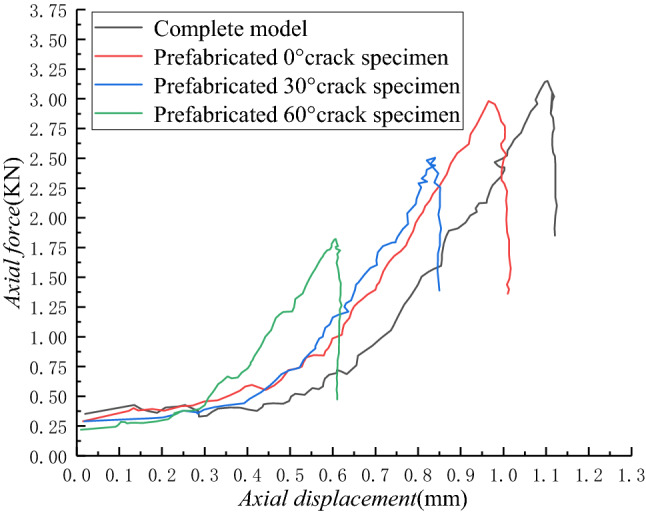
Axial force–displacement curves of different samples in the whole shear process.

The axial force–displacement curves of the whole shear process of different samples are shown in Fig. 4.

During the shearing process of the sample, its internal energy continued to accumulate until macroscopic crack damage occurred. Therefore, during the entire loading process, the area of load-longitudinal displacement can be used to determine the energy accumulated by the specimen^18^:

1 $$ A_{i} = \int_{0}^{a} {p_{i} da_{i} } $$

where *i* is the sampling point; *A*_*i*_ is the energy accumulated by the sample at a certain time, kJ; *P*_*i*_ is the stress of the sample at a certain time, MPa; and *a*_*i*_ is the displacement of the sample at a certain time, mm. For the integral in Eq. (1), the actual calculation can be based on the concept of the definite integral, using the load–displacement curve of the tested sample to calculate the area of the small trapezoidal strip area, that is:

2 $$ A_{i} = \frac{1}{2}\left( {p_{i + 1} + p_{i} } \right)\left( {a_{i + 1} - a_{i} } \right) $$

Due to the difference in the size of sandstone samples, the cumulative energy accumulated per unit of rupture area (called energy density W) is used to calculate according to the following equation:

3 $$ W = \frac{1}{RH}\sum\limits_{i = 1}^{n} {A_{i} } $$

where *W* is the peak energy density of the sample, kJ/m^2^. Energy analysis in this paper refers to energy per unit area, while *R* and *H* are the diameter and height of the sample, respectively, and the values in this paper are 50 mm and 25 mm.

The whole process of shearing in the sample is accompanied by the accumulation and release of energy. We use Eq. (3) to calculate the peak energy density *W* of different samples. Based on the analysis of elastic mechanics, under the action of the concentrated load on the radial disc specimen, tensile stress *σ*_*x*_ and compressive stress *σ*_*y*_ act on the diameter of the disc:

4 $$ \sigma_{x} = - \frac{2P}{{\pi RH}} $$

5 $$ \sigma_{y} = \frac{2P}{{\pi RH}}\left( {1 - \frac{{4R^{2} }}{{R^{2} - 4y^{2} }}} \right) $$

where *P* is chosen as the maximum force from the axial force–displacement curve, KN, and *y* is the distance from the center of the loading line, mm.

**Figure 5:**
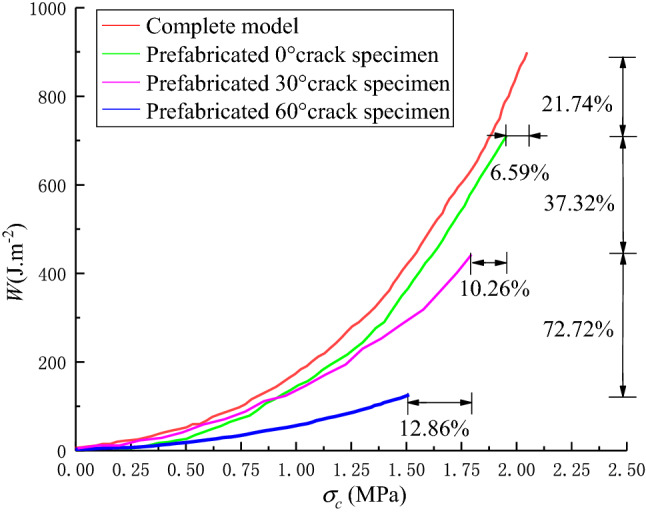
Relationship between peak energy density and tensile strength.

Based on the understanding that the tensile strength of rock materials is much lower than the compressive strength, the sample is split along the loading plane under tensile stress; therefore, the tensile strength of the sandstone samples can be calculated using Eq. (4). We then draw the relationship between the peak energy density (*W*) and the tensile strength (*σ*_*c*_), as shown in Fig. 5.

From Fig. 5, it can be seen that the peak energy density and tensile strength of different samples were different during the shearing process. Among them, the XS sample’s results are the largest, followed by the XS-0 sample, the XS-30 sample, and the XS-60 sample. The peak energy density and tensile strength of the XS-0 sample are only 21.74% and 6.59% lower than that of the XS sample. Combined with the crack propagation in Fig. 3, it can be seen that the prefabricated 0° crack had little effect on the mechanical properties of the sample, and the cracks generated by the two samples in the experiment are similar. With the increase of the prefabricated crack angle, the decreased degree of peak energy density and tensile strength gradually increased. Compared with the XS-30 sample, the reduction rate of the XS-60 sample increased to 72.72% and 12.86%, indicating that the crack influences the mechanical properties of the sample. The greater the inclination angle of the prefabricated crack, the worse the mechanical properties of the sample, and the stored energy and tensile strength decrease exponentially. On a macroscopic level, the derivation rate of the secondary cracks in the specimen gradually increase and approach the tip of the prefabricated cracks.

## PD theoretical model of brittle materials

Brittle materials refer to materials that have a macroscopic failure with only small deformations under loads such as in-situ stress or confining pressure, with little or no plastic deformation. At this stage, brittle materials are mainly studied in an idealized way: that is, the deformation that occurs is linear and irrecoverable^19^. This paper takes sandstone as the research object, so the bond-based PD theory and PMB model, which have unique advantages in the study of brittle materials, are introduced to study the change of internal energy in the whole process of rock material shearing.

### Bond-basis PD theory

Bond-based PD theory, as a special case of PD theory, considers that atoms are connected by "bonds" on the microscopic level of materials^20^. During the loading process of the material, the bonds begin to gradually deform. When the deformation of the bonds between two point pairs exceeds the critical elongation of the bonds, the bonds break, release energy, and there is no longer any interaction between the point pairs. When the cleavage of bonds accumulates to a certain extent, the material will undergo macroscopic damage^21^.

**Figure 6:**
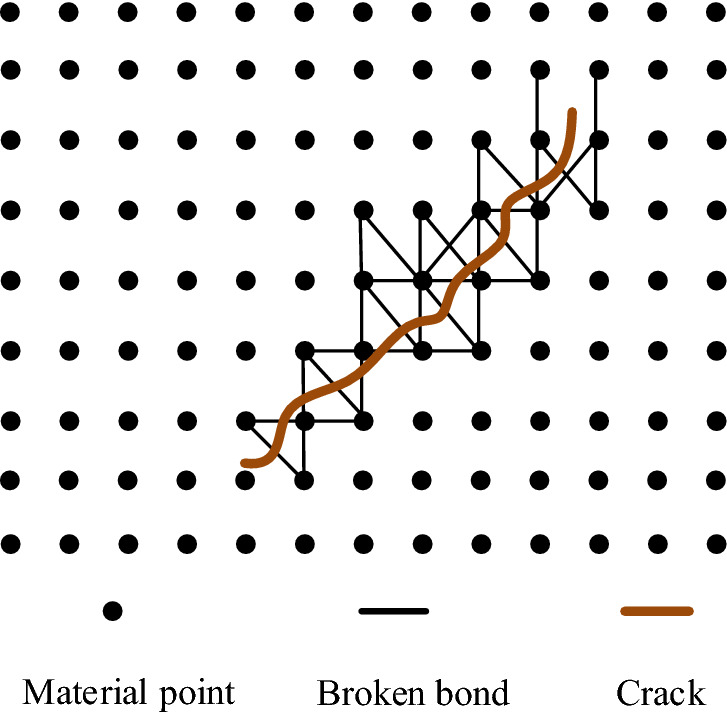
Bond-based PD theory.

Therefore, the bond-based PD theory is the overall combination of microscopic bond fracture and macroscopic damage, and its schematic diagram is shown in Fig. 6.

As seen in Fig. 6, the constitutive force expression of bond-basis PD theory can be obtained through the following equation:

6 $$ {\mathbf{f}}({{\boldsymbol{\upeta}}},{{\boldsymbol{\upxi}}}) = \frac{{({{\boldsymbol{\upeta}}},{{\boldsymbol{\upxi}}})}}{{\left| {{{\boldsymbol{\upxi}}} + {{\boldsymbol{\upeta}}}} \right|}}{\mathbf{f}}({{\boldsymbol{\upeta}}},{{\boldsymbol{\upxi}}}) = \frac{{({{\boldsymbol{\upeta}}},{{\boldsymbol{\upxi}}})}}{{\left| {{{\boldsymbol{\upxi}}} + {{\boldsymbol{\upeta}}}} \right|}}c({{\boldsymbol{\upxi}}})s $$

where **f** is the constitutive force function; $c({{\boldsymbol{\upxi}}})$ is a PD micro-modulus function, a second-order tensor; ${{\boldsymbol{\upxi}}}$ is the initial relative position vector of the material point; **η** is the relative position vector of the material point after loading; and *s* is the elongation of the bond, %.

In order to make the constitutive force function suitable for describing brittle materials, the elongation *s* of the bond is corrected, and the corrected value is:

7 $$ s = \frac{{{\mathbf{|\xi }} + {\mathbf{\eta |}} - {\mathbf{|\xi |}}}}{{{\mathbf{|\xi |}}}} $$

Using Eq. (7), the constitutive force function **f** can be modified, and the modified value is:

8 $$ {\mathbf{f}}({{\boldsymbol{\upeta}}},{{\boldsymbol{\upxi}}}) = \frac{{({{\boldsymbol{\upxi}}} + {{\boldsymbol{\upeta}}})}}{{\left| {{{\boldsymbol{\upxi}}} + {{\boldsymbol{\upeta}}}} \right|}}cs $$

where *c* is the elastic stiffness of the key and is a constant, called the 'micro-modulus', similar to spring stiffness. The micro-modulus *c* has different values in different situations, as shown in Table 2: where δ is the peri size of the material point *x*; *E* is the elastic modulus; *v* is the Poisson 's ratio; and *k* is the bulk modulus of three-dimensional space. *k'* is the bulk modulus of two-dimensional space, and its expression is:

9 $$ k^{\prime} = \frac{E}{3(1 - 2\mu )}\quad {\mathrm{Plane}}\;{\mathrm{stress}} $$

10 $$ k^{\prime} = \left\{ {\begin{array}{*{20}l} {E/2\left( {1 - v} \right)} \hfill \\ {E/2\left( {1 - v - 2v^{2} } \right)} \hfill \\ \end{array} } \right.\quad {\mathrm{Plane}}\;{\mathrm{strain}} $$

**Table 2:** Bond constant of *c* for bond-based PD.

Dimension	Poisson 's ratio(*v*)	Micromodulus(*c*)
3D	1/4	$\frac{{18k^{\prime}}}{{\pi \delta^{4} }}$
2D		
Plane strain	1/4	$\frac{{48k^{\prime}}}{{\pi \delta^{3} }}$
Plane stress	1/3	$\frac{{12k^{\prime}}}{{\pi \delta^{3} }}$
1D	0	$\frac{2E}{{\delta^{2} }}$

In the bond peridynamic constitutive model used in this paper, the material points interact only through pair potential energy. For isotropic and linear microelastic materials, this assumption leads to an effective Poisson's ratio of 1/3 for plane stress in 2D and 1/4 for plane stress in 3D.

To describe the damage to the material, the damage variable defined by former Soviet scholar Kachanov^22^ is introduced:

11 $$ D = \frac{{A^{*} }}{A} $$

where A* is the damage area of the specimen, m^2^, and *A* is the initial cross-sectional area of the specimen, m^2^.

By mathematically fitting the constitutive force function **f** with the damage variable D, we can obtain:

12 $$ D = \left\{ {\begin{array}{*{20}l} {1 - \exp \left( { - \frac{{s - s_{ec} }}{{s_{0c} }}} \right),} \hfill & {{\text{Nonlinear stage of compression zone:}} \quad s_{c} \le s \le s_{ec} } \hfill \\ {0,} \hfill & {{\text{Nonlinear stage of tension - compression zone:}} \quad s_{ec} \le s \le s_{et} } \hfill \\ {1 - \exp \left( { - \frac{{s - s_{et} }}{{s_{0t} }}} \right),} \hfill & {{\text{Non - linear stage of lashen District:}} \quad s_{et} \le s \le s_{t} } \hfill \\ {1,} \hfill & {\mathrm{Fracture:others}} \hfill \\ \end{array} } \right. $$

Perifield force and elongation and stress and strain are the relationships between force and deformation. Both of them describe the mechanical behavior of material failure, and their linear and nonlinear relationships should be basically consistent.

**Figure 7:**
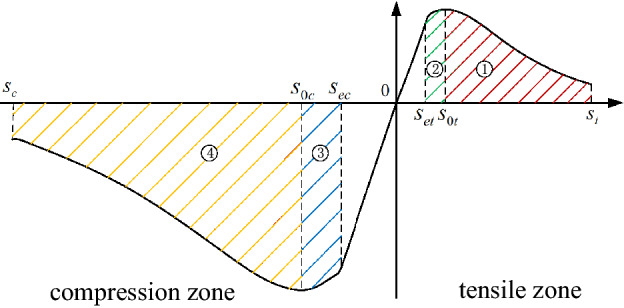
Damage constitutive force function curve of brittle materials.

Next, the constitutive force function of brittle materials was fitted with the full stress–strain curve of brittle materials, and the typical constitutive force function of brittle materials was obtained^23,24^, as shown in Fig. 7.

The linear segment describes the elastic deformation phase of the bond, without damage; the nonlinear segment describes the damage deformation phase of the bond, with nonlinear damage. *s*_*et*_ and *s*_*ec*_ are the linear elastic elongation when the bond is stretched and compressed, corresponding to the elastic deformation; *s*_*0t*_ and *s*_*0c*_ are the critical elongation when the bond is stretched and compressed, respectively, corresponding to the maximum value of the Perifield force; *s*_*t*_ and *s*_*c*_ are the elongation at cleavage when the bond is stretched and compressed, respectively, corresponding to the bond cleavage. The relationship between linear and nonlinear perifield force and elongation describes the bond cleavage process, which is consistent with the linear and nonlinear mechanical behavior of typical brittle material failure.

According to Fig. 7, the basic form of the constitutive force model with linear and nonlinear mechanical behavior can be obtained using:

13 $$ f(n,{{\boldsymbol{\upeta}}}) = \left\{ {\begin{array}{*{20}l} {cs(1 - D)\frac{{{{\boldsymbol{\upeta}}} + {{\boldsymbol{\upxi}}}}}{{{\mathbf{|\eta }} + {\mathbf{\xi |}}}},} \hfill & {s_{c} < s < s_{ec} \cup s_{et} < s < s_{t} } \hfill \\ {cs\frac{{{{\boldsymbol{\upeta}}} + {{\boldsymbol{\upxi}}}}}{{{\mathbf{|\eta }} + {\mathbf{\xi |}}}},} \hfill & {s_{et} < s < s_{et} } \hfill \\ {0,} \hfill & {others} \hfill \\ \end{array} } \right. $$

In Eq. (13), *s* can be obtained from **η** and ${{\boldsymbol{\upxi}}}$.

### PMB model of brittle materials

When using numerical simulation to solve problems using brittle materials, it is necessary to discretize the spatial region where the established model is located into multiple material points. The discretization process conforms to the formula PD discretization model. The solution method of the equation of motion is changed, and the integral is transformed into a summation. While formulating the solution, it is necessary to meet the stability requirements of brittle materials in order to ensure the convergence of the iterative process of the explicit difference method.

In the simulation calculation process of PD theory, the distance between the discrete material points and the size of the near-field range to be solved will affect the final solution accuracy. Therefore, when performing macro-scale simulation, the distance between the peri region radius δ and the material point Δx is when $\delta = 3.015\Delta x$ is satisfied, the calculation accuracy is better^25^.

Using the derived constitutive force model, other parameters are also transformed into a form that can be numerically calculated. The numerical calculation form of the deformation energy density W_PMB_ in the PMB material can be obtained as:

14 $$ W_{PMB} = \frac{{cs^{2} {{\boldsymbol{\upxi}}}}}{2}\Delta V_{j} $$

where W_PMB_ is the deformation energy density (hereinafter referred to as micropotential), and $\Delta V_{j}$ is the total volume of the point unit in the peri region, m^3^.

According to this calculation method, the relevant parameters and motion equations in the model can be integrated into four simple operations. The energy of the bonds between the material points can be calculated using these four operations, and there is no need to make continuity assumptions for the model.

## Numerical simulation of rock material shear

We next used the Python language to write a shear loading program for rock materials from the perspective of peridynamics. The program utilizes the LAMMPS atomic and molecular parallel simulator and establishes a meshless PD discrete model. After calculation, ParaView visualization software was used for post-processing to observe the specific deformation damage and energy distribution of the model.

The numerical model is a cylindrical specimen with a size of $\varphi 50{\mathrm{mm}} \times 25{\mathrm{mm}}$. Material parameters such as density, Poisson's ratio, and elastic modulus are all defined by numerical software. For the convenience of calculation, the total time step is set to 20,000 steps. In the simulation, it is assumed that the material points are uniformly distributed. As shown in Ref.^26^, the material point spacing of the discrete peridynamic is Δ=1mm, $\delta = 3.015\Delta$, so the simulated sample and the upper and lower press-heads are discretized into about 70,000 material points. According to the loading rate of rock mass in Refs.^27^ and ^28^, the lower boundary of the model is fixed, and the upper boundary is subjected to uniform load at the same speed of 0.1 mm/min as in the experiment. Due to the deformation and failure of the model under compression and shear, the main calculation data in this paper are as follows:

1. “compute all_stress all stress/atom NULL” is used to calculate the stress of atoms.
2. “variable strainx equal "(lx-v_L0)/v_L0" ”is used to calculate the strain of atoms.

Since the LAMMPS software directly removes the volume term in order to simplify the calculation, the “stress” calculated by the model itself has the unit of energy. Therefore, this paper directly utilizes the “stress” data to reflect the changes of energy within the model. Equation (13) is used as the constitutive force model of numerical simulation, and Eq. (14) is used as the energy calculation method to calculate the energy between bonds. The total energy is characterized by the average amount of energy released by the total bond fracture of the model.

**Figure 8:**
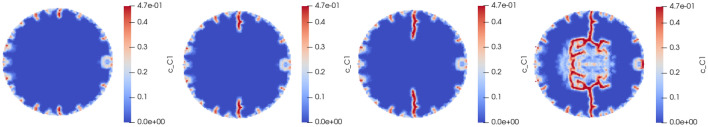
Shear energy change and damage of intact rock materials.

**Figure 9:**
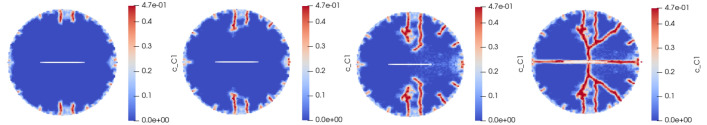
Shear energy change and damage of prefabricated 0° cracked rock materials.

**Figure 10:**
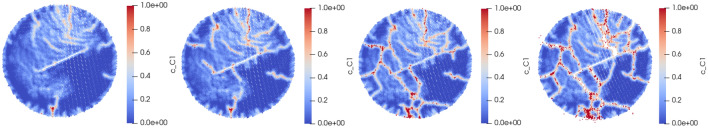
Shear energy change and damage of prefabricated 30° cracked rock materials.

**Figure 11:**
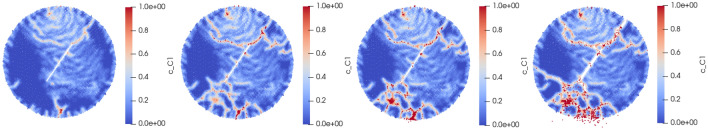
Shear energy change and damage of prefabricated 60° cracked rock materials.

In order to make the simulation results more accurate, the relaxation time was increased to relieve the initial stress and return the model to a balanced arrangement. In order to facilitate the comparison with the experimental results, the overall energy change and damage (c_C1) of the model are selected as the output results, and the simulation results are shown in Figs. 8, 9, 10 and 11.

As seen in Fig. 8, the model can be roughly divided into four stages for the whole process of shearing: the energy generation stage, the energy aggregation stage, the energy conduction stage, and the energy intersection stage. When the complete model is initially sheared, the energy starts to accumulate at the edge of the model, and then concentrates conduction at its longitudinal position. The deformation or fracture of the bond continues during the conduction process. When the model reaches a state of instability and failure, the energy gradually begins to accumulate in the middle of the model and then to disperse and conduct. The energy at both ends gradually gathers and spreads out in a grid pattern. This is why the XS sample shows a more deviated crack propagation state when it is destroyed in the experiment.

As seen in Fig. 9, the model with a prefabricated 0° crack will show the aggregation and conduction of two different energy bands of the complete model. In the beginning, the two groups' rate of energy conduction is roughly the same. With the continuous conduction of energy, the two groups gradually have a trend of convergence, but generally, they still show longitudinal conduction. When the model reaches the instability failure state, the two groups of energy begin to converge, and the internal energy begins to accumulate in the middle of the model, gradually affecting the tip of the prefabricated crack. In general, except for the appearance of two sets of energy bands, other states are similar to the complete sample; that is, when the prefabricated crack is 0°, the influence on the model is small, which is consistent with the macroscopic failure effect of the XS-0 sample in the experiment.

As seen in Fig. 10, the energy aggregation of the prefabricated 30° crack model begins to shift, not in the longitudinal direction, but at a certain angle to the tip of the prefabricated crack. When it finally intersects with the prefabricated crack, it is not at the crack tip position, but relatively close to it, which is consistent with the macroscopic damage effect of the XS-30 specimen in the experiment. The position of the prefabricated crack’s tip will also generate energy aggregation and gradually disperse to the whole model, but that accumulated energy is not enough to cause macroscopic damage to the sample, This is why the XS-30 sample shows only a partial secondary crack at the lower end of the model.

As seen in Fig. 11, the model energy of the prefabricated 60° crack is very small or almost not concentrated in the normal direction of the model. Only a small part of the energy is concentrated below the model, while a large area of energy aggregation begins to appear around the prefabricated crack. As the loading continues to increase, the energy accumulated at the tip of the prefabricated crack begins to follow the same direction and a perpendicular direction to the prefabricated crack. Meanwhile, the more obvious stress conduction on the lower surface perpendicular to the prefabricated crack, and the more obvious energy accumulated on the lower surface, gradually converge. The aggregation and conduction of energy in this way also leads to the secondary crack propagation pattern of the XS-60° specimen, connected to the crack tip and perpendicular to the prefabricated crack during failure.

**Figure 12:**
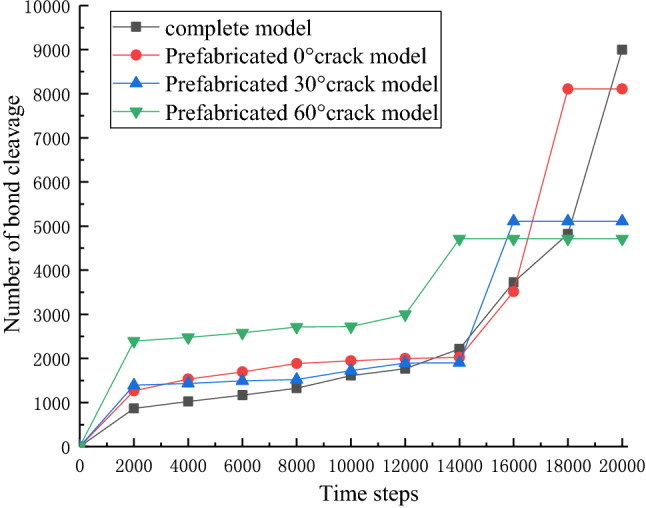
Number of bond cleavages per 1,000 steps for different models.

For different models, output the number of bond cleavage every 1000 steps, as shown in Fig. 12.

As shown by Fig. 12, throughout the whole shear process, the number of bond cleavages showed a trend of slowing down first and then steepening before they finally stabilized. Therefore, it can be assumed that the energy aggregation of the model in the process of shearing shows the law of slow aggregation first and then large aggregation. Different models have different time steps required for shear load failure and different numbers of bond cleavages during failure. Among them, the smallest difference in time step and number of bond cleavages is between the complete model and the prefabricated 0° crack model, and as the prefabricated crack inclination angle increases, the time step required for failure and the number of bond cleavages decrease significantly. The failure time step of the complete model is 20,000 steps, while the prefabricated 60° crack model starts to fail at 14,000 steps, and the number of bond cleavages during failure is only about half of the complete sample. It is thus shown that the prefabricated cracks have different degrees of influence on the shear damage of the model: the prefabricated 0° crack has a small influence, and the prefabricated 60° crack has a greater influence, which is consistent with the experimental conclusion.

In addition, when the model is initially sheared, the energy accumulated by different models is different. The prefabricated 60° crack model has the largest initial energy accumulation, and as the prefabricated crack inclination angle decreases, the initial energy accumulation is also smaller. This indicates that the larger the angle of the crack, the greater the damage of the model at the initial stage of loading. After calculation, the energy released by bond cleavage after shear failure in different models is shown in Table 3.

**Table 3:** Energy released by bond cleavage in different models.

Type of model	Released energy(eV)
Complete model	1.392 × 10^5^
Prefabricated 0°crack model	1.290 × 10^5^
Prefabricated 30°crack model	1.122 × 10^5^
Prefabricated 60°crack model	0.818 × 10^5^

**Figure 13:**
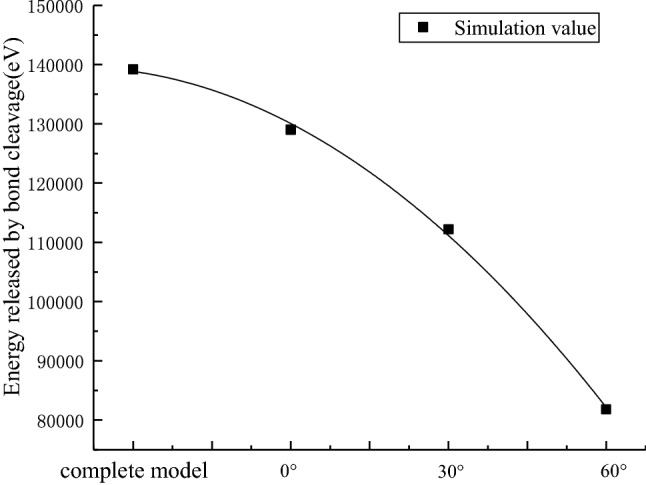
Energy curves released by bond cleavage in different models.

The energy curves released by the different models are plotted in Fig. 13, according to the data from Table 3.

As seen in Fig. 13, as the prefabricated crack inclination angle increases, the energy released by the bond fracture gradually decreases exponentially in different models. This is consistent with the decreasing trend of the peak energy density in the experiment, indicating that the experimental results are true and effective. The inclination angle of the prefabricated crack has a great influence on the tensile strength and energy release of the sample, which has great reference significance for rock mechanics.

## Conclusion

In this paper, the fracture characteristics and energy change laws of sandstone materials under the action of prefabricated crack angles were studied. The whole shear process of rock samples with different dip angles was analyzed using peridynamics combined with laboratory experiments from the perspective of energy. This study can provide theoretical support for underground engineering construction, such as judging the tendency of rock mass shedding or impact around the roadway in roadway support, or judging whether coal and rock will collapse in the process of underground coal mining. Its results can effectively predict the occurrence of underground accidents, prevent casualties, and reduce equipment damage and costs. The results show that:

1. Prefabricated cracks will weaken the tensile strength and peak energy density of the specimen. When the prefabricated crack inclination angle is 0°, the weakening effect is small. With the increase of the prefabricated crack inclination angle, the weakening effect increases exponentially, and the strength of the specimens will show significant differences.
2. The angle of the prefabricated crack will affect the initiation and propagation of the experimental crack. When the prefabricated crack angle is 0°, the crack propagation is roughly the same as that of the intact specimen. With the increase of the prefabricated crack inclination angle, the specimen’s crack initiation position gradually shifted to the crack tip.
3. During the whole shearing process of the sample, the number of bond cleavages increase continuously, showing the law of first slowness and then sharpness. With the increase of the prefabricated crack inclination angle, the number of bond cleavages continuously decrease. When the prefabricated 60° crack sample failed, the number of bond cleavages was only about 5,000 pairs.
4. Based on the splitting damage model established by the simulation software, the fracture of the bond and the released energy are mainly concentrated in the position of the upper and lower indenters and the tip position of the prefabricated crack. The energy in the whole shearing process of the complete model shows nearly linear aggregation in the longitudinal direction. With the increase of the prefabricated crack inclination angle, the energy will gradually start to accumulate at the crack tip and be transmitted to the loaded position, eventually resulting in macroscopic fracture. Calculating the stored energy of different cracked rock masses under load can effectively predict the impact tendency of the rock mass, and can provide a reference for the prediction of mine support and rock bursts.

## Data Availability

The data used to support the findings of this study are included within the article.
