# Editorial Expression of Concern: Study on mechanical properties and energy change of rock materials in whole splitting process based on peridynamics

**DOI:** 10.1038/s41598-026-64877-y

**Published:** 2026-08-05

**Authors:** Hewan Li, Jian Liu, Laigui Wang, Tianjiao Ren

**Affiliations:** College of Mechanics Engineering, Liaoning Technical University, No. 47, Zhonghua Road, Xihe District, Fuxin City, 123000 Liaoning Province China

Editorial Expression of Concern to: *Scientific Reports* 10.1038/s41598-023-30394-5, published online 24 February 2023.

The Editors are issuing this Expression of Concern.

Following publication, concerns were raised about inaccuracies in Equations 7, 12, and 13. In equation 7, η and ξ are presented as vectors and should therefore be formatted in bold. In equation 12, where D = 0, the term 'Non-linear’ should be ‘Linear’ stage of the tension-compression zone. Additionally, Non-linear stage of the ‘lashen’ District should be the non-linear stage of 'tension’ District. Furthermore, references 9 and 11 appear to be translated from Chinese and their existence could not be verified.

The Readers are therefore advised to take caution while interpreting this content.

The Authors did not respond to the Editors’ communication about this Editorial Expression of Concern.

